# Colquhounia Root Tablet Protects Rat Pulmonary Microvascular Endothelial Cells against TNF-*α*-Induced Injury by Upregulating the Expression of Tight Junction Proteins Claudin-5 and ZO-1

**DOI:** 10.1155/2018/1024634

**Published:** 2018-11-18

**Authors:** Wenjie Zhou, Guocui Shi, Jijia Bai, Shenmao Ma, Qinfu Liu, Xigang Ma

**Affiliations:** ^1^Department of Critical Care Medicine, General Hospital of Ningxia Medical University, Yinchuan, Ningxia 750004, China; ^2^Department of Respiratory Medicine, Cangzhou People's Hospital, Cangzhou, Hebei 061000, China; ^3^Resident Standardized Training Base, General Hospital of Ningxia Medical University, Yinchuan, Ningxia 750004, China

## Abstract

**Background:**

There are currently limited effective pharmacotherapy agents for acute lung injury (ALI). Inflammatory response in the lungs is the main pathophysiological process of ALI. Our preliminary data have shown that colquhounia root tablet (CRT), a natural herbal medicine, alleviates the pulmonary inflammatory responses and edema in a rat model with oleic acid-induced ALI. However, the potential molecular action mechanisms underlining its protective effects against ALI are poorly understood. This study aimed to investigate the effects and mechanism of CRT in rat pulmonary microvascular endothelial cells (PMEC) with TNF-*α*-induced injury.

**Methods:**

PMECs were divided into 6 groups: normal control, TNF-*α* (10 ng/mL TNF-*α*), Dex (1×10^−6^ M Dex + 10 ng/mL TNF-*α*), CRT high (1000 ng/mL CRT + 10 ng/mL TNF-*α*), CRT medium (500 ng/mL CRT + 10 ng/mL TNF-*α*), and CRT low group (250 ng/mL CRT + 10 ng/mL TNF-*α*). Cell proliferation and apoptosis were detected by MTT assay and flow cytometry. Cell micromorphology was observed under transmission electron microscope. The localization and expression of tight junction proteins Claudin-5 and ZO-1 were analyzed by immunofluorescence staining and Western blot, respectively.

**Results:**

TNF-a had successfully induced an acute endothelial cell injury model. Dex and CRT treatments had significantly stimulated the growth and reduced the apoptosis of PMECs (all* p* < 0.05 or 0.01) and alleviated the TNF-*α*-induced cell injury. The expression of Claudin-5 and ZO-1 in Dex and all 3 CRT groups was markedly increased compared with TNF-a group (all* p* < 0.05 or 0.01).

**Conclusion:**

CRT effectively protects PMECs from TNF-*α*-induced injury, which might be mediated via stabilizing the structure of tight junction. CRT might be a promising, effective, and safe therapeutic agent for the treatment of ALI.

## 1. Introduction

Acute lung injury (ALI) is a major cause of acute respiratory failure with high morbidity and mortality in critical care medicine [1]. ALI is characterized with persistent pulmonary inflammation and increased microvascular permeability [1]. The inflammatory response in the lungs is the main pathophysiological process of ALI. TNF-*α* is an important factor mediating the inflammatory response during injury, which initiates inflammatory cascade and destroys the alveolar capillary barrier [2], leading to increased permeability and pulmonary edema. The pulmonary microvascular endothelial cell (PMEC) barrier acts as the first line of defense against inflammatory factor attack and plays a key role in the development of lung injury. The tight junction among PMECs, composed of transmembrane proteins including Claudins, Occludin, and ZO-1, serves as an important structure controlling pericellular permeability and regulating PMEC barrier functions [3].

Although the pathophysiology and treatments of ALI have been investigated in numerous studies, there are currently limited effective pharmacotherapies agents. Glucocorticoids have been commonly used to treat lung injury induced by various causes [4–6]. Dexamethasone (Dex) is a widely used synthetic glucocorticoid compound with proven protection effects against ALI [7, 8]. Moreover, Dex has been frequently used as positive control in several pharmacological studies on potential therapeutic agents for ALI [9]. However, these hormone therapies may trigger a variety of adverse reactions, such as infection, elevated blood glucose, peptic ulcer, and osteoporosis [10]. Therefore, it is urgent to search for novel therapeutic reagents for ALI. The traditional Chinese medicine colquhounia root contains several alkaloids, terpenoids, lactones, and phenolic acids, such as triptolide and epicatechin [11]. Colquhounia root has several beneficial pharmacological properties, such as anti-inflammatory, immunosuppressive, antitumor, and analgesic activities [12–14]. Recent studies have found that colquhounia root attenuates allergic encephalomyelitis in rats by reducing capillary permeability during inflammation and decreasing inflammatory exudate [15]. Our preliminary data have shown that colquhounia root alleviates the pulmonary inflammatory responses and edema in rat model with oleic acid-induced ALI [16]. However, the potential molecular action mechanisms underlining its protective effects against ALI have not been investigated. In this study, we treated rat PMECs with different doses of colquhounia root and explored its protective effects and potential mechanism on the cells and intercellular tight junctions using Dex as a treatment control. Our findings shall shed insight on potential novel therapeutic reagents against ALI.

## 2. Material and Methods

### 2.1. Reagents

Colquhounia root tablets (CRT, 0.18 g/tablet) were purchased from the Pharmaceutical Factory of the Chongqing Academy of Chinese Materia Medica (Chongqing, China). A CRT was ground into powders, dissolved in 1 mL of dimethyl sulfoxide (DMSO), and filtered through a 0.22-um sterile filter. TNF-*α* (400-14, purity ≥ 98%, Peprotech, Rocky Hill, NJ, USA) was prepared into 0.1 mg/mL stock. Dex (Dexamethasone, D4902, purity ≥ 97%, Sigma, Shanghai, China) was prepared into 0.03185 M stock. All stocks were stored at -20°C until use.

### 2.2. High-Performance Liquid Chromatography (HPLC)

The major active components of CRT were determined by HPLC. Triptolide was purchased from the Chinese National Institute for Food and Drug Control and dissolved in 60% methanol for 10 *μ*g/mL standard solution for HPLC. Finely ground CRT powder (3.6 g) was mixed with 50 mL of methanol, processed using an ultrasonic cleaner (KQ-250DE, Kunshan Ultrasonic Instrument, China) at 250 w, 50 KHz for 1 h, and filtered. The residue was washed twice with 10 mL of methanol. The methanol solution was combined and loaded on neutral alumina column (length: 300 mm, inner diameter: 1.5 cm). The column was eluded with 60 mL of acetone. The eluate was collected, dried, dissolved in 5 mL of 60% methanol, and filtered through a 0.45-*μ*m millipore filter. Samples of the filtrate were injected into an InertSustain C18 chromatographic column (4.6 x 250 mm, 5 *μ*m) in a LC-20AT HPLC system (Shimadzu, Tokyo, Japan). HPLC was performed using the following parameters: mobile phase: acetonitrile-water (25:75); detection wavelength: 220 nm; flow rate: 1 mL/min; column temperature: 35°C; injection volume: 10 *μ*L. The number of theoretical plates of the triptolide peak should be no less than 2000.

Epicatechin was purchased from the Chinese National Institute for Food and Drug Control and dissolved in 60% methanol for 100 *μ*g/mL standard solution for HPLC. CRT powder (0.9 g) is mixed with 40 mL of water-saturated ethyl acetate, processed by an ultrasonic cleaner (250 w, 50 KHz) for 30 min, filtered, and dried. The residue was dissolved in 10 mL of 60% methanol. Aliquots of samples and standard solution (20 *μ*L) were injected onto an InertSustain C18 column and HPLC was performed using the following parameters: mobile phase: 0.63% glacial acetic acid solution-methanol-acetonitrile (82.5:2:15.5); detection wavelength: 280 nm; flow rate: 1 mL/min; column temperature: 35°C.

### 2.3. Cells and Grouping

Rat PMECs were purchased from the Bioleaf Biotech Inc. (Shanghai, China) and cultured in RPMI-1640 medium (Gibco, Rockville, MD, USA) supplemented with 5% fetal bovine serum (FBS, Gibco), 100 *μ*g/mL streptomycin and 100 U/mL penicillin penicillin/streptomycin (Invitrogen, Shanghai, China) at 37°C, 5% CO_2_ in an incubator. Cells were seeded into 96-well plates at a density of 1×10^5^ cells/well and incubated overnight. Cells were then divided into the following 6 groups and cultured for an additional 48 h for subsequent examinations: normal control group without any treatment, TNF-*α* group cultured in medium containing 10 ng/mL TNF-*α*, Dex group in 1×10^−6^ M Dex and 10 ng/mL TNF-*α*, CRT high group in 1000 ng/mL CRT and 10 ng/mL TNF-*α*, CRT medium group in 500 ng/mL CRT and 10 ng/mL TNF-*α*, and CRT low group in 250 ng/mL CRT and 10 ng/mL TNF-*α*.

### 2.4. MTT Cell Proliferation Assay

Cell proliferation in all groups was analyzed using MTT assay kit (KeyGen Biotech Inc., Nanjing, China). Briefly, cell medium was discarded and cells were incubated with 90 *μ*L of FBS-free medium and 20 *μ*L of MTT at 37°C for 4 h. Cells were treated with 150 *μ*L of DMSO for 10 min. The optical density (OD) was detected with a microplate reader under the wavelength of 490 nm. Three wells were prepared for each group. The cell proliferation inhibition rate was calculated using the following formula: cell proliferation inhibition rate (%) = (OD value in control group - OD value in experimental group)/OD value in control group × 100%.

### 2.5. Cell Apoptosis Assay

Cell apoptosis in all groups was analyzed using Annexin V-FITC apoptosis detection kit (Bestbio Biotech Inc., Shanghai, China). In brief, cells were collected upon the completion of treatment. Cells were washed twice with PBS, resuspended in 400 *μ*L of 1× binding buffer, stained with 5 *μ*L of FITC Annexin V in the dark at 4°C for 15 min. Cells were then incubated in 10 *μ*L of PI in the dark for 5 more min. The fluorescence of cells was analyzed by a BD Accuri C6 flow cytometry (BD Biosciences, Franklin Lakes, NJ, USA) at 488 nm within 1 h.

### 2.6. Transmission Electron Microscopic Observation

After treatment, cells in all groups were collected, washed twice with 0.1 M natrium cacodylate buffer solution, and fixed in 2% glutaraldehyde solution at 4°C for 5 min. Cells were collected and incubated in 2% glutaraldehyde solution at 4°C for 1 h. Cells were collected and incubated 3 times in 0.1 M natrium cacodylicum buffer solution at 4°C for 30 min each. Cells were then incubated in 1% osmic acid for 1 h, washed twice with 0.1 M natrium cacodylicum buffer solution for 15 min each, dehydrated with 30%, 50%, 70%, 80%, 90%, and 100% ethanol, infiltrated with epoxypropane for 15 min, and treated with embedding solution at 35°C for 6 h. Embedded cells were cut into 50-nm slices using an ultrathin slicer, stained in 3% silver citrate solution, and observed under an H7650 transmission electron microscope (Hitachi, Japan).

### 2.7. Immunofluorescence Staining

Normal PMECs at exponential phase were inoculated into a six-well plate with sterile slides and incubated at 37°C, 5% CO_2_ for 24 h. The cells were washed twice with prewarmed PBS, fixed with 4% paraformaldehyde solution for 20 min at room temperature, and washed 3 times with PBS. Cells were blocked with 5% BSA for 1 h at room temperature and incubated with primary antibody (mouse anti-rat Claudin-5, 1:50; rabbit anti-rat ZO-1, 1:50, Invitrogen, USA) overnight at 4°C. Cells were washed 3 times with PBS and incubated with FITC-labeled secondary antibody (goat anti-rabbit IgG, 1: 100; goat anti-mouse IgG, 1:100, Zhongshan Golden Bridge Biotech Inc., Beijing, China) for 1 h at room temperature in the dark. Cells were stained with DAPI. The slide was sealed and observed under an Olympus FV1000 confocal microscope (Olympus, USA).

### 2.8. Quantitative Reverse Transcription PCR (qRT-PCR)

The expression of Claudin-5 and ZO-1 mRNA was determined by qRT-PCR. Briefly, total RNA was extracted from cells using TRIzol reagent (Invitrogen, USA). Reverse transcription was performed using reverse transcription kit (Takara, Japan). The primers were designed and synthesized by Genscript Inc. (Nanjing, China): Claudin-5 forward 5'-CAGCGTTGGAAATTCTGGGTC-3', reverse 5'-ACACTTTGCATTGCATGTGCC-3'; ZO-1 forward 5'-TGGTGCTCCTAAACAATC-3', reverse 5'-TGCTATTACACGGTCCTC-3'; and *β*-actin forward 5'-CCCATCTATGAGGGTTACGC-3', reverse 5'-TTTAATGTCACGCACGATTTC-3'. The qRT-PCR reaction mixture was prepared using real time PCR kit (TaKaRa, Japan): SYBR Premix Ex Taq II 10 *μ*L, forward primer 0.8 *μ*L, reverse primer 0.8 *μ*L, cDNA template 2 *μ*L, and dH_2_O 6.4 *μ*L. Reaction was performed on an ABI PRISM 7500 Fluorescent Quantitative PCR System (Applied Biosystems, Foster City, CA, USA) used according to the following reaction conditions: 95°C 5 s followed by 45 cycles of 95°C 5 s, 57°C (Claudin-5)/60°C (ZO-1) 30 s and 72°C 40 s. The experiment was performed in triplicate and the expression level was calculated using the 2-ΔΔCt method. *β*-actin was used as the internal control.

### 2.9. Western Blot

Total protein was extracted from cells using total protein extraction kit (Keygen Biotech, Nanjing, China) and quantified using BCA protein assay kit (Keygen Biotech). Equal aliquots (20 *μ*g) of protein were separated by SDS-PAGE and transferred to polyvinylidene difluoride membranes. The membranes were blocked with 5% skim milk for 2 h and incubated with primary antibodies (mouse anti-rat Claudin-5, 1:500, Invitrogen, USA; rabbit anti-rat ZO-1, 1:250, Invitrogen, USA; mouse anti-rat *β*-actin, 1:500, Zhongshan Golden Bridge Biotech.) overnight at 4°C. The membrane was washed 3 times with 1 x TBST and incubated with HRP-conjugated secondary antibody (goat anti-rabbit IgG, 1:5000; goat anti-mouse IgG, 1:5000, Zhongshan Golden Bridge Biotech.) at room temperature for 2 h. The immunoreactivity was detected using the NCI 5079 ECL detection system (Themo Fisher, USA). The image was analyzed by ChemiGenius Bioimaging System (Syngene, MD, USA) and the relative expression of proteins was quantified using Image J (National Institutes of Health, Bethesda, USA) with *β*-actin as the internal reference.

### 2.10. Cell Transfection

siRNAs were designed and synthesized by Genscript Biotech. (Nanjing, China): si-Claudin-5 forward: 5'-GUCCGGGAGUUCUAUGAUCCA-3', reverse: 5'-GATCATAGAACTCCCGGACTA-3'; si-ZO-1 forward: 5'-UGUUGAACAUGCUUUUGCUGT -3', reverse: 5'-AGCAAAAGCAUGUUCAACATT -3'; si-nagetive control (NC) forward: 5'-UUCUCCGAACGUGUCACGUTT-3', and reverse: 5'-ACGUGACACGUUCGGAGAATT-3'. Normal PMECs at 70% confluence in 6-well plates were transfected with 30 nM of siRNAs or siNC using Lipofectamine 3000 transfection agent (Invitrogen, Shanghai, China) according to the manufacturer's instructions. Cells were then incubated in medium containing 500 ng/mL CRT and 10 ng/mL TNF-*α*. After 48 h, cells were collected and the expression of Claudin-5 and ZO-1 was determined by Western blot as described above. Cell proliferation and apoptosis were also measured as described above.

### 2.11. Statistical Analysis

Data were expressed as mean ± standard deviation. Data analysis was performed using SPSS 17.0 statistical software (IBM SPSS, Chicago, IL, USA). Difference among groups was analyzed by one-way analysis of variance (ANOVA) followed by post hoc SNK-q test. Rates were compared by chi-square test.* p *< 0.05 was considered statistically significant.

## 3. Results

### 3.1. Major Components of CRT

**Figure 1:**
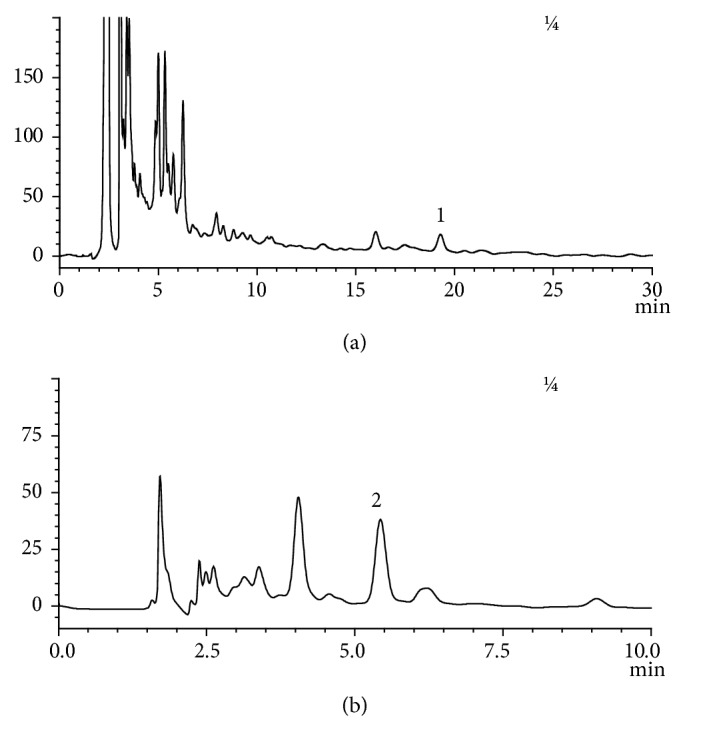
**HPLC analyses of the major active components of CRT**: triptolide (a) and epicatechin (b) at the wavelengths of 220 nm and 280 nm, respectively.

As shown in Figure 1, HPLC data suggests that each tablet (0.18 g) contains 3.04 *μ*g of triptolide (C20H24O6) and 0.13 mg of epicatechin (C35H14O6).

### 3.2. CRT Stimulates the Growth of PMECs

**Figure 2:**
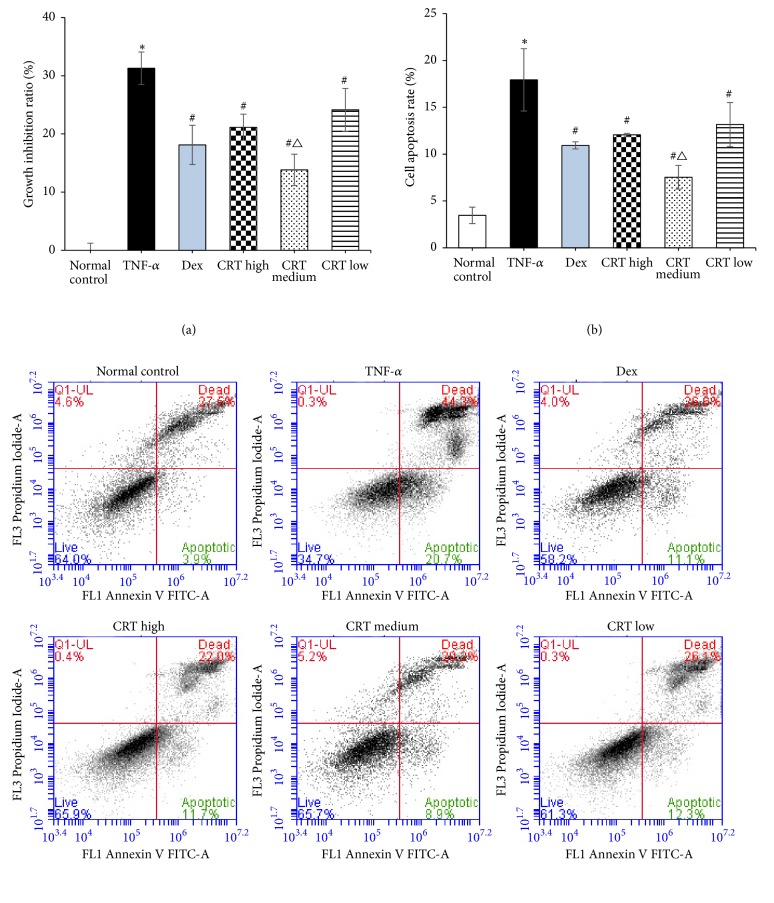
**CRT treatment inhibited the cell proliferation inhibition rate and apoptosis rate induced by TNF-** **α**. Rat PMEC monolayers were divided into 6 groups: normal control, TNF-*α*, Dex, CRT high, CRT medium, and CRT low groups. After 48 h incubation, cell proliferation (a) and apoptosis (b) were detected by MTT assay and flow cytometry, respectively. The cell proliferation inhibition rate (%) = (OD value in control group - OD value in experimental group)/OD value in control group × 100%. *∗*, P<0.05, TNF-*α* group vs. normal control group; #, P<0.05 treatment groups vs. TNF-a group; △, P<0.05, CRT groups vs. Dex group.

The growth of PMECs was compared by MTT assay. As shown in Figure 2(a), the TNF-*α* had significantly increased the growth inhibition rate of PMECs compared with normal control group (*p* = 0.016). The growth inhibition rate of PMECs in Dex and all CRT groups was significantly lower than that in TNF-*α* group (*p *= 0.033, 0.045, 0.020, and 0.039, respectively). The growth inhibition rate in CRT medium group was significantly lower compared with Dex group (*p* = 0.032), whereas the other two CRT groups had similar rate as that in Dex group (*p* > 0.05).

### 3.3. CRT Reduces the Apoptosis of PMECs

Further, the effects of CRT on the apoptosis of PMECs were evaluated by flow cytometry. As shown in Figure 2(b), the apoptosis rate in TNF-*α* group was significantly higher than that in normal control group (*p* = 0.026). The apoptosis rate of PMECs in all 4 treatment groups was significantly reduced compared with TNF-*α* group (*p* = 0.023, 0.037, 0.019, and 0.042, respectively). The apoptosis rate in CRT medium group was significantly lower compared with Dex group (*p* = 0.028), but the other two CRT groups had similar rate as that in Dex group (*p* > 0.05).

### 3.4. CRT Alleviates the TNF-*α*-Induced Cell Injury

**Figure 3:**
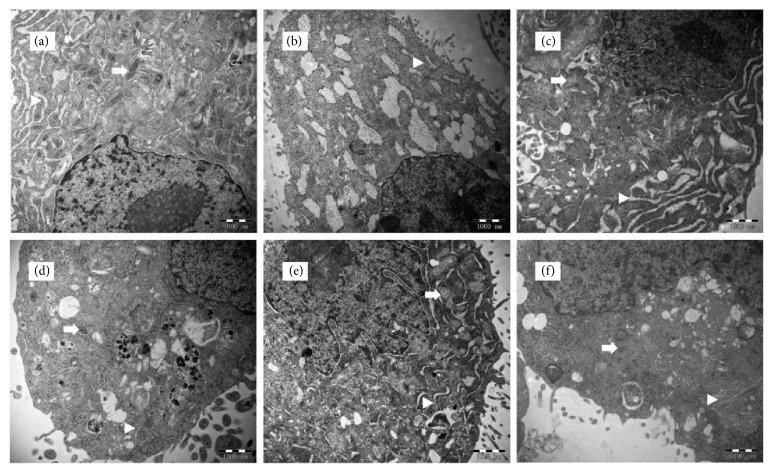
**CRT alleviated the TNF-** **α** **-induced cell injury. **Rat PMEC monolayers were divided into 6 groups: normal control (a), TNF-*α* (b), Dex (c), CRT high (d), CRT medium (e), and CRT low groups (f). After 48 h incubation, intracellular microstructures were observed under an H7650 transmission electron microscope (20000×). Arrows and triangles mark the mitochondria and endoplasmic reticulum, respectively.

The effect of CRT on the ultrastructure of PMECs was observed under transmission electron microscope (Figure 3). In normal control group, there are abundant cytoplasms with a number of intact organelles (mitochondria, endoplasmic reticulum, and Golgi) and few pinocytotic vesicles. After the treatment of TNF-a, cells exhibited severe injuries. The number of organelles was obviously decreased. Mitochondria and endoplasmic reticulum swelling were clearly observed. Mitochondria cristae disappeared, and abundant vacuole-like structures were formed. Nuclear fragmentation/lysis and cell apoptosis were observed in some cells. In Dex and the 3 CRT groups, cells exhibited more normal morphology and less severe damage compared with TNF-*α* group. Although mitochondrial swelling was still noticed, mitochondrial structure was intact. The number of vacuoles in the cytoplasm was greatly reduced. CRT medium group had clearly much more intracellular organelles and less vacuoles than the other two CRT groups, indicating lighter cell injury.

### 3.5. CRT Upregulates the Expression of Tight Junction Protein Claudin-5 and ZO-1

**Figure 4:**
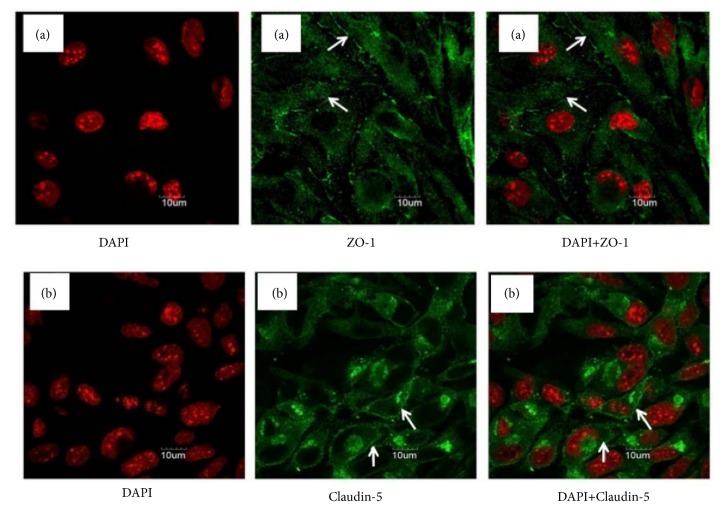
**Analysis of the localization of tight junction proteins ZO-1 (a) and Claudin-5 (b) in normal rat PMECs by immunofluorescence assay. **Normal rat PMECs were subjected to DAPI and immunofluorescence staining. Cells were observed under a confocal microscope. Arrows indicate the linear fluorescence of Claudin-5 and ZO-1 along the endothelial cell membrane, suggesting that both proteins are localized at the edge of endothelial cells. Abundant diffused fluorescence was also detected among the cells, suggesting that both proteins coordinately form the intracellular tight junction structure.

**Figure 5:**
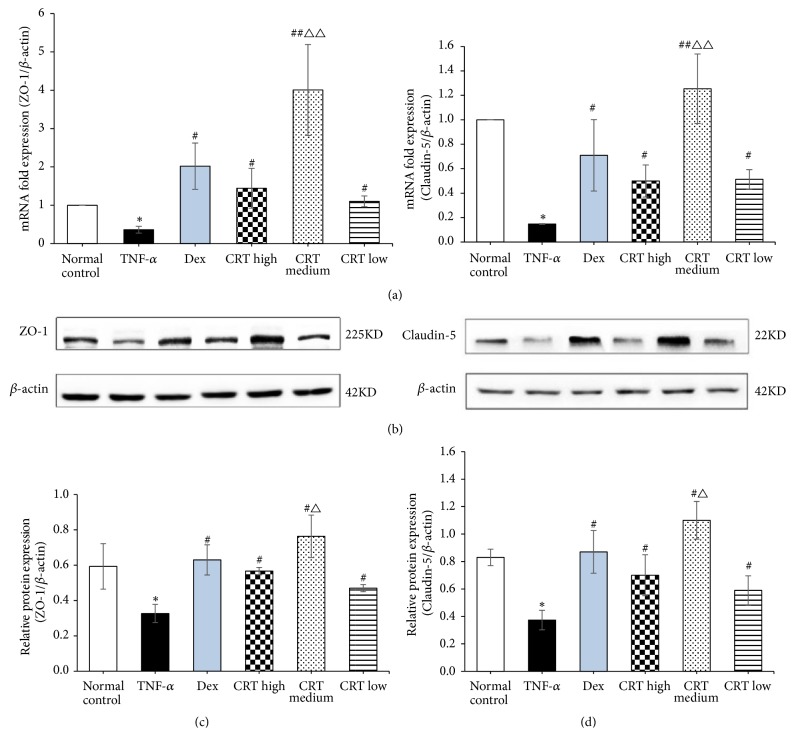
**CRT upregulates the expression of tight junction proteins Claudin-5 and ZO-1. **Rat PMEC monolayers were divided into 6 groups: normal control, TNF-*α*, Dex, CRT high, CRT medium, and CRT low groups. After 48 h incubation, the expression of Claudin-5 and ZO-1 mRNA (a) and protein (b) was detected by qRT-PCR and Western blot, respectively. *∗*, P<0.05, *∗∗*, P<0.01, TNF-*α* group vs. normal control group; #, P<0.05, ##, P<0.01, treatment groups vs. TNF-a group; △, P<0.05, △△, P<0.01, CRT groups vs. Dex group.

**Figure 6:**
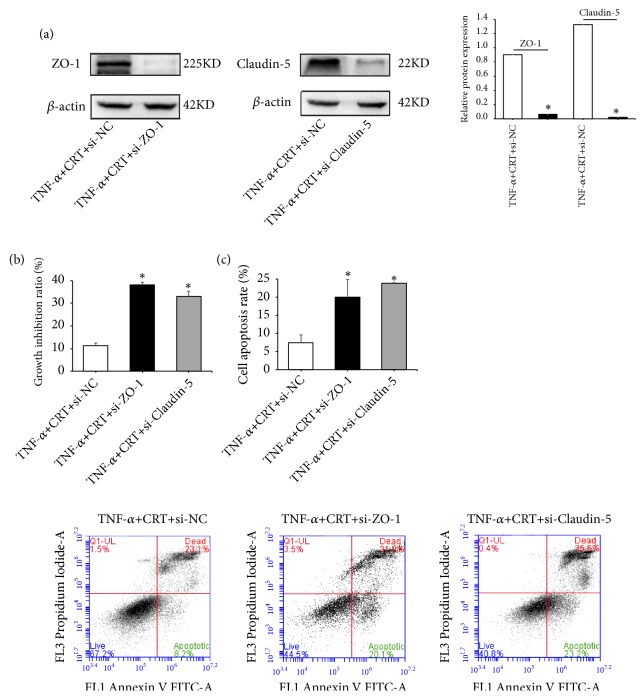
**Therapeutic effects of CRT were greatly reduced by siRNAs. **Rat PMECs at 70% confluence were transfected with siRNAs or siNC. Cells were incubated in medium containing 500 ng/mL CRT and 10 ng/mL TNF-*α*. After 48 h, cells were collected and the expression of Claudin-5 and ZO-1 was determined by Western blot (a). Cell proliferation (b) and apoptosis (c) were also measured. *∗*, p < 0.05, TNF-*α*+CRT+si-ZO-1 or si-Claudin-5 vs. TNF-*α*+CRT+si-NC.

The localization of tight junction proteins Claudin-5 and ZO-1 was detected by immunofluorescence assay using a confocal microscope. As shown in Figure 4, linear fluorescence staining of Claudin-5 and ZO-1 was observed along the endothelial cell membrane, indicating that both proteins are localized at the edge of endothelial cells. Furthermore, abundant diffused fluorescence was also detected among the cells, which suggested that both proteins coordinately form the intracellular tight junction structure. To clarify the action mechanism of CRT against TNF-a-induced cell injury, we further examined the expression of Claudin-5 and ZO-1 mRNA and protein in different groups. It was found that the expression of ZO-1 and Claudin-5 mRNA in TNF-a group was significantly lower compared with normal control group (*p* = 0.034 and 0.008, respectively, Figure 5(a)). Claudin-5 and ZO-1 mRNA expression in Dex and the 3 CRT groups was remarkably increased compared with TNF-*α* group (all* p* < 0.01 or 0.001). Claudin-5 and ZO-1 mRNA expression in CRT medium group was significantly higher compared with Dex group (*p *≦ 0.001 and 0.004), whereas the other two CRT groups had similar rate as that in Dex group (*p *> 0.05). Consistently, the expression of Claudin-5 and ZO-1 protein showed similar change pattern as the mRNA expression (Figure 5(b)). To further confirm the therapeutic effects of CRT mediated by upregulating Claudin-5 and ZO-1 expression, PMECs were transfected with siRNAs targeting Claudin-5 or ZO-1 and incubated in medium containing TNF-*α* and CRT. Western blot results showed ZO-1 and Claudin-5 expression was successfully downregulated by siRNAs (*p* = 0.032 and 0.015, respectively, Figure 6(a)). The cell proliferation inhibition and apoptosis rate in TNF-*α*+CRT+si-ZO-1 and TNF-*α*+CRT+si-Claudin-5 group was significantly higher compared with TNF-*α*+CRT+si-NC group (all* p *< 0.05, Figures 6(b)-6(c)), suggesting that the silencing of ZO-1 and Claudin-5 expression had blocked the therapeutic effects of CRT. In other words, the protecting effects of CRT were mediated via modulating the Claudin-5 and ZO-1 expression.

## 4. Discussion

TNF-*α* released early during ALI acts on PMECs through blood circulation, which damages cells and alveolar capillary barrier, and thus leading to lung injury [17, 18]. In this study, TNF-a was added to the cell culture medium to mimic the biological condition when ALI was developed. Electron microscope results showed that TNF-a treatment has damaged organelle structure and induced mitochondrial and endoplasmic reticulum swelling. Mitochondrial crista nearly disappeared, and abundant vacuole-like structures were observed. Moreover, TNF-a group had significantly inhibited the growth and stimulated the apoptosis of PMECs, suggesting that TNF-a had successfully induced an acute endothelial cell injury model.

In the current study, the main components of colquhounia root tablets were identified by HPLC as triptolide and epicatechin. Extensive studies have reported the anti-inflammatory [19, 20], immunosuppressive [21, 22], antitumor [23, 24] effects of triptolide and epicatechin. Studies have shown that triptolide and epicatechin have protection effects against lung injury [25, 26]. Although triptolide and epicatechin are the two major components of CRT, the herbal medicine contains several other active ingredients such as alkaloids, terpenoids, and lactones. Moreover, in clinical practice, CRT has been widely used to treat nephrotic syndrome and rheumatoid arthritis [27]. Our preliminary study has found that CRT can effectively alleviate pulmonary edema [28]. Therefore, instead of focusing on the pharmacological effects of individual components, we investigated the role and mechanism of CRT in protecting lung injury aiming to provide a basis for its clinical application in ALI treatment. Our data showed that CRT had significantly lighter mitochondrial and endoplasmic reticulum swelling and lower number of intracellular vacuoles. Intact organelle structures were observed under electron microscope. Furthermore, cells in CRT groups had exhibited higher cell proliferation and lower apoptosis rate as compared with TNF-*α* group, suggesting that CRT had effectively protected cells from TNF-a-induced injury.

Tight junction is an intercellular junction complex that is widely present in the blood-brain barrier, intestinal barrier, retinal barrier, glomerular basement membrane barrier, and alveolar capillary barrier [29–33]. It plays a key role in regulating the transport of water and solute molecules and maintaining tissue permeability [34, 35]. Claudins are important structural molecules in tight junction. Claudin-5 is strongly expressed in PMECs and regulates paracellular permeability [36]. The overexpression of Claudin-5 in PMECs and cerebral vascular endothelial cells reduces permeability of tight junction and thus protects endothelial barrier function [36, 37]. Studies have found that reduced Claudin-5 expression in endothelial cells results in a rapid increase in the permeability of pulmonary blood vessels [36]. As the key structure of tight junction, ZO-1 directly affects the pulmonary barrier permeability. When ZO-1 expression is inhibited, transepithelial electrical resistance of mouse PMECs is markedly decreased, leading to increased pulmonary permeability and impaired lung barrier functions [38]. The intracellular parts of Claudin-5 and ZO-1 interact with each other to maintain the stability of tight junction structure. In this study, immunofluorescence assay demonstrated linear fluorescence staining of both Claudin-5 and ZO-1 along the endothelial cell membrane and abundant diffused fluorescence among these cells, suggesting both proteins form the intracellular tight junction structure and coordinately regulate the paracellular permeability.

Studies have suggested that TNF-*α* can downregulate the expression of several tight junction proteins in the lungs of mice, including Claudin-2, -4, -5, and ZO-1, increasing the lung barrier permeability [39]. Consistently, our results showed a significant decrease in the intracellular expression of Claudin-5 and ZO-1 mRNA and protein in TNF-*α* group, indicating that TNF-*α* destroyed the integrity of tight junction by inhibiting the expression of structural proteins. In contrast, Claudin-5 and ZO-1 expression in CRT groups was significantly enhanced when compared with TNF-*α* group, suggesting that the protective CRT was mediated via stabilizing the structure of tight junction and endothelial barrier. It is worth noting that CRT medium group had higher therapeutic effects than Dex group. The action mechanism of Dex is mediated through the pituitary-adrenal system. Dex regulates the expression of anti-inflammatory genes by binding to the glucocorticoid receptor (GR) [40]. In contrast, CRT does not exert the anti-inflammatory effect via the pituitary-adrenal system [41], but instead reduces oxidative stress and inflammation by regulating NF-*κ*B signaling pathway [42]. The NF-*κ*B pathway directly regulates tight junctions, and its activation increases paracellular permeability and impairs barrier function [43, 44]. TNF-*α* can affect the inflammatory response by activating the NF-*κ*B pathway and thus increase the pericellular permeability [45]. Therefore, we speculate that the higher therapeutic effects of CRT medium group than Dex group may be associated with its modulation on the NF-*κ*B pathway.

One may also notice that the CRT medium group exhibited significantly lower growth inhibition rate and apoptosis rate compared with CRT high and low groups. Electron microscopic image also revealed much lighter destruction of PMECs in CRT medium group compared with the other two CRT groups. Furthermore, the highest Claudin-5 and ZO-1 mRNA and protein expression was observed in CRT medium group among the three CRT groups. Altogether, our results suggested that the medium dose of CRT exerted the best therapeutic effects. Studies have shown that high-dose CRT exhibits cytotoxic effects and inhibits cell proliferation [13, 14]. In our preliminary test, we also found that high-dose CRT inhibited the proliferation of alveolar type II epithelial cells. Therefore, the CRT high group in the current study had lower therapeutic effects compared with CRT medium group. It is thus important to find the optimal CRT treatment dose in clinical practice.

## 5. Conclusion

In summary, this study has found that CRT effectively reduces the TNF-*α*-induced growth inhibition rate and apoptosis rate of PMECs. The protective effects of CRT against TNF-*α*-induced injury might be mediated via stimulating the expression of Claudin-5 and ZO-1 in PMECs, which stabilizes the structure of tight junction and endothelial barrier. As a natural herbal medicine, CRT might be a promising effective and safe therapeutic agent to substitute the glucocorticoids for the treatment of ALI. Future studies are needed to investigate the signaling mechanism involved in the regulation of CRT on tight junction proteins.

## Data Availability

The data used to support the findings of this study are available from the corresponding author upon request.
